# VALIDA project: Validation of allergy *in vitro *diagnostics assays (Tools and recommendations for the assessment of *in vitro* tests in the diagnosis of allergy)

**DOI:** 10.1515/almed-2020-0051

**Published:** 2020-08-21

**Authors:** María L. Casas, Ángel Esteban, Miguel González-Muñoz, Moisés Labrador-Horrillo, Mariona Pascal, Aina Teniente-Serra

**Affiliations:** Clinical Analysis Service, Fundación Alcorcón University Hospital, Alcorcón, Madrid, Spain; Spanish Society of Laboratory Medicine (SEQC-ML), Barcelona, Spain; Clinical Analysis Service, University General Hospital of Alicante, Alicante, Spain; Immunology Service, La Paz University Hospital, Madrid, Spain; Spanish Society of Immunology (SEI), Barcelona, Spain; Allergology Service, Vall d’Hebron University Hospital, Barcelona, Spain; Spanish Society of Allergology and Clinical Immunology (SEAIC), Madrid, Spain; Immunology Service, CBD, Hospital Clínic de Barcelona, IDIBAPS, Universitat de Barcelona, Barcelona, Spain; ARADyAL Research Network, Carlos III Institute, Madrid, Spain; Immunology Service, LCMN, Germans Trias i Pujol University Hospital, Badalona, Spain

**Keywords:** allergy, diagnosis *in vitro*, immunoglobulin E, recommendations

## Abstract

*In vitro* allergen-specific immunoglobulin E (IgE) detection and quantification tests are routinely performed in clinical laboratories to diagnose patients with a suspected allergy. Numerous commercial assays are available to test for allergies, but the results can vary widely, thereby influencing both diagnosis and treatment. Given the challenges posed by differences in the various assays for *in vitro* determination of specific IgE, a group of experts has compiled in a document a series of recommendations on the implications that the use of a certain *in vitro* technique may have and the impact on the management of the allergic patient that the differences between the various techniques represent. The reading and analysis of this consensus document will help to understand the implications of the change of *in vitro* diagnostic method in the management of the patient with allergy, in the quality of life and in the socioeconomic costs associated with the disease.

## Introduction

The discovery of immunoglobulin E (IgE) and the development of the first *in vitro* diagnostic test to determine the presence of this antibody have had a profound impact on the diagnosis of IgE-mediated allergic diseases [1].

Allergy is a highly prevalent disease [2], [3] and its impact on quality of life (QoL) can be significant. Allergies may also have high costs—both direct and indirect—for national health care systems and for society [4], [5].

Currently, in the Spanish market several different commercial assays are available for *in vitro* determination of total (tIgE) and specific IgE (sIgE). Given the wide range of available tests, together with the complexity of diagnosing and treating this disease, and the increasing prevalence [6], [7], [8], [9], there is a clear need to establish evidence-based recommendations based on an objective evaluation of currently available tests [10].

The aim of the present document is to summarize the evidence on commercially available *in vitro* diagnostic tests for allergies, and to provide recommendations to help guide selection of the most appropriate tests for use in routine clinical practice, and the factors to consider when switching from one assay to another.

## Materials and methods

The contents of this document were developed by an expert working group, comprised by six specialists in immunology and allergy. The working group was appointed by the three main societies involved in allergy diagnosis: the Spanish Society of Laboratory Medicine (SEQC-ML), the Spanish Society of Immunology (SEI), and the Spanish Society of Allergy and Clinical Immunology (SEAIC). Each society elected two of its members as its representatives, forming a panel of six experts.

Several key questions that needed to be addressed in the document were elaborated. To do so, a bibliographic search was performed to identify articles on the following key topics: 1) differences among the various *in vitro* sIgE assays; 2) the characteristics of the ideal *in vitro* test for sIgE; 3) recommendations from the main clinical guidelines that address allergy diagnosis, and 4) possible consequences of switching diagnostic tests on patient management [4], [11], [12], [13], [14], [15]. In order to quantify the scientific evidence, a bibliographic search of the existing publications for each of the different *in vitro* sIgE detection techniques commercially available in Spain was carried out. The search was carried out in the Medline database using the following English terms for each of the *in vitro* techniques: (“Allergy AND Immunology” [Mesh] OR Allergy) AND (ImmunoCAP NOT ISAC), (“Allergy AND Immunology” [Mesh] OR Allergy) AND (ImmunoCAP AND ISAC), (“Allergy AND Immunology” [Mesh] OR Allergy) AND (Immulite), (“Allergy AND Immunology” [Mesh] OR Allergy) AND (Euroline), (“Allergy AND Immunology” [Mesh] OR Allergy) AND (Allergy Explorer-ALEX). No additional filters were applied to the search. Subsequently, publications not corresponding to the above search criteria were manually removed and the number of publications, by technique and by year, from the publication of the first paper of each technique to the present time was collected.

Clinicaltrials.gov database was searched to identify all completed clinical trials of these same *in vitro *diagnostic tests. The following search terms were used: “name of test”, “allergic condition or disease”. We then selected the completed studies. The search parameters included studies performed between January 1, 1989 (the year that the first reagent for the detection of tIgE and sIgE was marketed) and October 1, 2019.

Finally, the expert group met again to discuss the key questions investigated in the bibliographic search, and to compare these findings to the experts’ personal clinical experience. Next, the experts sought to reach a consensus agreement about the recommendations to be included in this document.

## Importance of *in vitro* testing to diagnose allergies

According to the guidelines of the European Academy of Allergy and Clinical Immunology (EAACI) [7], the recommended diagnostic protocol for a patient with suspected allergy starts with a complete medical history, the findings of which direct the subsequent sensitization study, typically an *in vivo* skin prick test (SPT) followed by an *in vitro* serum analysis.

*In vivo* allergy testing, usually an SPT, is fast and highly sensitive and specific. However, most patients require both *in vivo* and *in vitro* tests due to the limitations of these tests. The main limitations of SPT are lack of standardized extracts, which results in inter-test variability, the potential for subjectivity in the interpretation of results, which affects reproducibility, the infrequent—but not impossible—risk of inducing a systemic allergic reaction, the inability to assess recombinant allergenic components and, the need to postpone testing in patients with certain skin conditions (dermographism, dermatitis, urticaria) and in those taking antihistamines or other drugs [16].

Given the drawbacks mentioned before, *in vitro* testing has assumed an increasingly important role in the diagnosis of allergies in the last decade. This diagnostic approach increases the likelihood of making a diagnosis, with a greater diagnostic specificity, while reducing risks to the patient arising from a possible cross-reactivity or clinical symptoms that do not match the sensitization data obtained in the laboratory [17].

In recent decades, the molecular diagnosis of allergy has have been extensively documented. The consensus statement published by WAO-ARIA-GA^2^LEN task force [10] states that *in vitro* diagnosis plays an important role in three key phases of allergy diagnosis: 1) differentiation between genuine sensitization and cross-reactivity; 2) risk assessment for new systemic reactions in selected food allergies, and 3) identification of the optimal candidates for immunotherapy. Accordingly, clinicians need to be aware of diagnostic differences between the various *in vitro* testing techniques.

To better understand the most important concepts related to *in vitro* detection of sIgE for the diagnosis of allergies, it is important to be familiar with the most common terminology [18]:

### *In vitro* determination of IgE

Quantification of serum IgE concentration in a blood sample of a subject. This approach can be used to determine tIgE, regardless of specificity—the sIgE to a given extract or allergenic component.

### Allergen

An allergen is an antigen capable of inducing an immune response, which stimulates the production of sIgE antibodies in a predisposed organism after initial contact. Subsequently, this gives rise to an antigen-antibody reaction that can trigger clinical symptoms if a new exposure occurs (hypersensitivity reaction type I, or allergic reaction). Allergens are the antigens of the allergic response.

### Total extract

A complete allergen extract is an aqueous or glycerinated solution or lyophilized protein obtained, in most cases, by aqueous extraction from a complete allergenic source (e.g., peanut or olive pollen).

### Allergen components

These are the individual components of an allergenic source that react with the sIgE. Most of these are proteins with the capacity to trigger an allergic reaction. Thus, an allergenic source (e.g., a food or pollen) should be considered a mixture of different allergenic “components”.

### Component-resolved diagnosis (CRD)

Component-resolved diagnosis (CRD), also called molecular diagnosis, involves the detection and quantification of sIgE antibody levels for a given individual component. The CRD provides a much more accurate diagnosis and can also be used to assess the risk of new reactions and to identify the optimal candidates for therapeutic purposes.

## Differences between the available *in vitro* diagnostic tests

*In vitro* sIgE detection techniques are based on the binding of a given allergen to a solid or liquid phase, to which the patient’s sIgE for that allergen will be bound. IgE molecules not specific to the allergen in question will be removed by washing. Subsequently the sIgE-allergen complex will be incubated with a labeled anti-IgE antibody that will allow detection of these allergens. The signal emitted by the labeled antibody will allow the measurement of the concentration of IgE [19]. Immunoassays for sIgE require a standard calibration curve to determine the amount of sIgE present in the patient’s serum, which is calibrated according to the total IgE standard established by the International Reference Preparation for Human IgE of the World Health Organization [20]. This is used to interpolate results at kUA/L of sIgE, where one unit equals 2.4 ng of IgE. There is evidence that one unit (kUA/L) of sIgE is equivalent to one unit (kU/L) of tIgE [21].

In recent years, technological advances, together with a growing demand for *in vitro* diagnostic assays, have led to the development of several new testing methods based on the IgE detection system described in the previous paragraph. These tests include the following, all of which are available on the Spanish market: ImmunoCAP™ and ImmunoCAP™ ISAC (Thermo Fisher Scientific); Immulite^®^ (Siemens); Euroline^®^ (Euroimmun); and ALEX^®^/ALEX2^®^ (Macro Array Diagnostics).

Published analyses of the characteristics of these tests show that, although all the assays are based on antigen-antibody recognition, they differ in allergen binding methods, signal detection methods, the required sample volume, type of quantification, and degree of automation [22], [23], [24], [25]. Two different systems can be used to perform the *in vitro* allergy test: singleplex assays, which detect sIgE levels for a single specific allergen or allergenic source, or multiplex assays, which assess for the presence of sIgE against a battery of allergens, simultaneously [26].

Here we specify the characteristics of each of these systems:

### Singleplex systems

The ImmunoCAP™ assay has a portfolio of more than 600 allergens (including more than 100 molecular components), which are quantitatively determined using fluoroenzyme immunoassay.

The Immulite^®^ assay includes more than 480 different allergenic extracts and 33 molecular components. Immulite uses a chemiluminescence enzyme immunoassay, providing quantitative results.

### Multiplex systems

The ImmunoCAP™ ISAC multiplex assay uses a fixed matrix of 112 recombinant or purified native allergen components fixed in triplicates, with a semiquantitative (fluorescence) determination.

The ALEX^®^/ALEX2^®^ assay uses a fixed matrix of more than 120 allergenic extracts and 170 molecular components, providing a semiquantitative measure of total IgE and quantitative results for sIgE, using solid phase immunoassay technology (colorimetry).

Euroline^®^ is an assay capable of testing for approximately 100 different allergen panels (including total extracts and components), yielding semi-quantitative results based on solid phase immunoassay technology (colorimetry).

Table 1 summarizes the main characteristics of these assays.

**Table 1:** Comparison of the available *in vitro* diagnostic tests for allergies.

*In vitro* diagnostic method	Supplier	Complete extracts	Components	Calibration curve	Reading method	Process	Vol. (µL) 70	iCCD	Detection range [70]	Method/results
ImmunoCAP™	Thermo Fisher Scientific	>600	101	Yes	Fluorimetry	Automated	40	No	0.10–100 kU_A_/L	Singleplex/quantitative
Immulite^®^	Siemens	>400	33	Yes	Chemiluminescence		50	No	0.10–100 kU_A_/L	
ImmunoCAP™ ISAC	Thermo Fisher Scientific	0	112	Yes	Fluorimetry	Not automated	30	No	0.3–100 kU_A_/L	Multiplex/semi-quantitative
ALEX^®^	Macro Array Diagnostics	>120	>170	Yes	Colorimetry		100	Yes^a^	sIgE: 0.3–50 kU_A_/L	Multiplex/Total IgE, semiquantitative
									tIgE: 1–2500 kU/L	Specific IgE, quantitative
Euroline^®^	Euroimmun	92 panels, from 2 to 50 components per panel		No	Colorimetry		100–400	Yes	0.35–100 kU_A_/L	Multiplex/semi-quantitative

^a^1/5 dilution. Characteristics of the *in vitro* diagnostic tests available in the Spanish market. These data were obtained from the specifications described in the product data sheet for each test. Vol: volume; iCCD: CCD inhibition.

All diagnostic allergy tests must provide an optimal balance between sensitivity and specificity and be supported by a strong scientific evidence. Ideally, the test should also cover a wide range of allergens and allow for automated testing. According to Crameri et al., ImmunoCAP should be considered the gold standard *in vitro* diagnostic assay [28] until new scientific evidence becomes available for the other tests. In fact, correlation studies use the ImmunoCAP as the standard of reference to compare with other assays [27,^29^,30].

## Considerations before switching to a different *in vitro* assay

### Correlation between tests

The published data indicate that the results of the various *in vitro* tests are not comparable or interchangeable [23], [31], [32], [33]. Although there may be some degree of correlation between tests, the results are not interchangeable through conversion factors due to the lack of units referred to a common standard [23], [33], [34], [35], [36], [37]. For example, Wood et al. [35] compared Immulite to ImmunoCAP, demonstrating that none of the currently validated *in vitro* diagnostic tests for allergies reliably correlate with the others and that the data obtained with a given assay cannot be reproduced with another testing technique. Given the important clinical repercussions of sIgE quantification, the choice of one method or another is a relevant decision.

### Sources of allergens: quality and reproducibility

The quality of the allergens used in the different assays can vary for several different reasons: the season in which the raw material is collected (e.g., pollens); the system used to preserve the material; the degree of difficulty in identifying the allergen; contamination with other allergenic sources, which may result in cross-reactivity; and differences in the extraction technique (recombinant production versus purification) [38]. For all these reasons, the allergens should be subjected to rigorous quality control.

It is also important to know if the allergen contains cross-reactive carbohydrate determinants (CCDs) which are oligosaccharides present in many allergens with high reactivity and little clinical relevance, which can lead to false positives. With respect to the prevalence of these antigens and their impact, it is estimated that they may present reactivity in 7.5–35% of the patients, so it can be a problem in the diagnosis [39], [40], [41]. Three strategies are currently applied to avoid false positives by CCDs: 1) recombinant production of allergens (in the case of components), 2) use of CCD inhibitors during the sIgE detection process, or 3) use of MUXF3 (an allergen composed exclusively of carbohydrate epitopes present in many plant glycoproteins) as a positivity control for CCDs. With the use of these strategies there is a decrease in false positives and therefore an increase in diagnostic accuracy [41], [42], [43].

### Portfolio diversity

A varied allergen portfolio is important, especially about the total number of allergen extracts and molecular components, as both provide complementary data to ensure an accurate diagnosis. *In vitro* allergy tests reveal not only the allergenic source that patients are sensitized to, but also the specific allergenic components within this source, which provides valuable additional clinical information [44, 45].

In routine clinical practice, the value of a broad portfolio of allergens, which allows clinicians to diagnose more patients, is crucial given the highly variable and often complex clinical pathology of allergic disease [46]. This is especially relevant in our region (Spain)—and in other Mediterranean countries, such as Italy and Greece—where polysensitization is a serious problem [47].

### Differences in the scientific literature

It is important to be aware of the differences between the available assays. This can be done by evaluating the published evidence to make an objective decision regarding the most suitable technique.

Before selecting a specific *in vitro* diagnostic assay, we must first ensure that there is sufficient scientific evidence to support the clinical use of that test, keeping in mind that the assay’s purpose is to provide reliable data to improve clinical management of the patient. The use of tests not supported by a robust evidence base could result in a delayed and less accurate diagnosis, which in turn could have a negative impact on the patient’s quality of life.

For this reason, a bibliographic search of the published scientific literature on the various *in vitro* sIgE detection tests was conducted. The results are detailed below.

**Figure 1:**
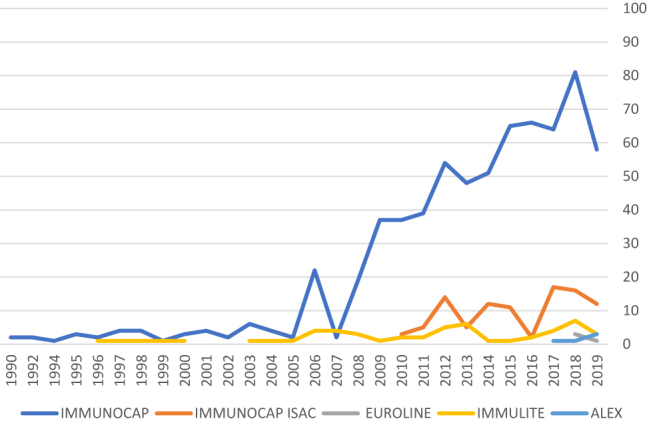
Chart of publications on Medline by *in vitro* diagnostic technique and by year.

Following the chronological order of appearance of evidence for each *in vitro* diagnostic technique for allergy, and using the search criteria described above (see methodology section), it can be seen that at the time of writing this document ImmunoCAP (1990) has more than 600 publications in the Medline database, Immulite (1996) has been referenced in 52 indexed articles, ImmunoCAP ISAC (2010) appears in 117 articles, Euroline (2018) in seven articles, and ALEX (2018) is the technique used in three publications (Table 2, Figure 1). Due to the impact of geographic location on sensitization profiles, beyond assessing the number of publications, it is also very important to have local evidence: ImmunoCAP and ImmunoCAP ISAC are the techniques with the most evidence so far (Supplementary material Table 1). ImmunoCAP and ImmunoCAP ISAC have more than 45 and 12 completed studies in Spain, respectively. Immulite has been used in two national studies (Supplementary material, Table 1 and 2).

**Table 2:** Number of published studies on the various *in vitro* diagnostic tests.

Technique	Total number of publications in Medline^a^	Publication rate^b^	Clinical studies (total number of patients included)
ImmunoCAP^®^	633	22.1	22 (2381)
Immulite^®^ 2000	52	2.16	1 (102)
ImmunoCAP ISAC^®^	117	13	0
Euroline^®^	7	1.4	1 (235)
ALEX^®^	3	1.5	0

^a^The number of completed clinical studies on allergies reported in https://clinicaltrials.gov. ^b^Total publications per year since first publication.

### Impact of switching between *in vitro* diagnostic methods

When assessing the impact of changing the method of determining sIgE *in vitro*, it should always be considered that a positive test only indicates sensitization, and only if it is associated with clinical reactivity (symptoms) can we speak of allergy [48]. There is no universal cut-off points for the different allergens that allow prediction of the probability of clinical reactivity associated with a given value of sIgE, so these must be validated clinically for each technique. If a laboratory changes the assay, the reference values must be re-established, since the previous ones will no longer be valid. In these cases, the laboratory must communicate with the clinician to determine how to make the transition. In addition, after switching, it will be necessary to review how to apply the test results in routine clinical practice [23], [29], [32].

### Impact on the initial diagnosis of the patient

Differences between diagnostic tests may affect the interpretation of the results and cause confusion between the protocols followed by laboratories and clinical services that manage the allergic patient. For example, the cut-off values for an oral provocation test based on experience with a given *in vitro* diagnostic test are not transferable to the values used for other assays for clinical decision-making. For example, food provocation tests are based on local cut-off points, with each center establishing its own threshold levels; in these cases, if the center decides to switch to a new technique, then the cut-off points will have to be re-established [23]. This has several implications: increased diagnosis-related costs, patient inconvenience, risk to patient safety, and the potential to overload the health care system.

### Impact on therapeutic decision making

At present, the only treatment proven to modify the natural course of allergic disease is desensitization, known as allergen-specific immunotherapy (SIT). Studies have shown that SIT can prevent disease progression, and the therapeutic effects persist even after the treatment regimen has been completed [49]. As its name implies, SIT is specific to a given allergenic extract. Consequently, it is essential to accurately identify the disease-causing allergen in order to select the most appropriate treatment prescription.

For this reason, determination of the sIgE against total extract, complemented by molecular diagnosis, can improve patient selection for SIT, such as in allergies to pollen [50, 51] and *Hymenoptera *venom [52]. In this regard, it is important to have a single method that can determine the components and total extracts, thus permitting differentiation between allergic patients who have multiple genuine sensitizations from those with multiple sensitizations due to cross-reactivity [53], especially in countries with high prevalence of polysensitized patients [54]. In addition, *in vitro* diagnosis also makes it possible to evaluate the risk of an adverse reaction to immunotherapy through the use of allergenic sources, such as olive pollen in areas with high pollen exposure, since sensitization to certain allergenic components (e.g., Ole e 9, and Ole e 7) is associated with this type of reaction [55,56].

### Impact on follow-up

In the course of follow-up of the allergic patient, changing the *in vitro* diagnostic assay during follow-up may be more problematic than it would be for a patient with an initial diagnosis. Indeed, many studies have made this same recommendation, suggesting that—due to the variability between assays (e.g., Immulite and other sIgE testing methods)—it is preferable to use the same assay to monitor the course of disease [27], [34], [37], [51].

Similarly, switching tests is not recommended in patients with food allergies who require a food tolerance test after a period of avoidance, as the various tests are all based on different sIgE cut off points [57].

### Cost-related impact of test selection in allergic disease

When selecting a diagnostic test and treatment for allergies (or any illness), a key consideration is cost-effectiveness. This implies that centers must base their decision on both technical criteria and costs (normally the unit price of the test) when selecting the assay. This approach, within the framework of public procurement, allows health professionals to assess which of the available methods provides the best cost to benefit ratio (cost-effectiveness).

Specialists who work in immunology and allergology must understand the technical characteristics of the available tests, and the differences between them, in order to fully understand the implications of switching tests and the costs (both direct and indirect) of this decision [58,59].

To date, published cost-effectiveness studies focus on the performance of ImmunoCAP ISAC and SPT. In 2016, NICE analyzed four studies involving them in a systematic review [60]. Hermanasen et al. (2012, 2013) compared the cost effectiveness of ImmunoCAP ISAC against double-blind controlled oral provocation and SPT in children with peanut allergy. A 5-year Markov model was used, with ImmunoCAP ISAC being the most efficient against provocation and SPT [61], [62]. Glaumann et al. (2013) also studied the cost effectiveness of ImmunoCAP ISAC against oral provocation and SPT, again showing ImmunoCAP dominance over the other techniques [63]. Furthermore Hermansson et al. (2012) [64] and Mascialino et al. (2013) [65] analyzed the cost-effectiveness of ImmunoCAP ISAC against SPT in a pollen-allergic population using a Markov model with a 9-year horizon and found that the sum of ImmunoCAP ISAC and SPT reduces the prescription of immunotherapy against SPT alone.

## Discussion

At present, European legislation does not require clinical trials to validate medical devices (unlike drugs) prior to marketing authorization, in contrast to the United States, where the Food and Drug Administration requires clinical trials for such devices [66] thus differing from drugs. The only requirement for commercialization in Europe is that the manufacturer obtains the CE marking certificate, which requires a comparative study with another device, and must also show that these results are reproducible over time. The results of this comparison must be included with the product instruction leaflet. This is relevant to the *in vitro* analysis of sIgE given the nature of IgE versus other analytical parameters, which is why clinicians and hospitals need to be very familiar with the various *in vitro *diagnostic tests for sIgE when selecting the best therapeutic option.

The consequences of an imprecise diagnosis are wide ranging, potentially impacting several different areas, which includes financial implications:

Laboratory. The time and expense needed to conduct a comprehensive clinical validation of a new *in vitro* diagnostic test must be considered. As explained above, this validation does not require clinical trials involving patients prior to commercialization. This legislative requirement means that it is the laboratory’s responsibility—and, by extension, all those involved in the diagnosis of the allergic patient—to design and carry out the clinical validation, which should take into account the specific characteristics of the population reality in the laboratory influence area to confirm that the new diagnostic method meets the necessary requirements, both in terms of the allergen panel and the sensitivity and specificity.
Clinical. Any change in the diagnostic test for allergies could lead to inconclusive results if new reference values have not been established which requires additional resources (i.e., funds) to compare the results. This implies higher costs (more tests and compensation for the clinician’s time).
Logistics. If the new allergy test does not have a sufficiently broad portfolio of allergens, in many cases the clinician will need to outsource sIgE determination, with the corresponding expense and potential delay in test results.
  Although we have mainly discussed only the direct costs of switching, there may be additional clinical consequences (e.g., patient quality of life) and indirect costs, which could be considerable [56].
  On the other hand, it should be noted that the lack of head-to-head comparison studies between the different techniques has been a limitation in the preparation of this manuscript. Furthermore, not only are head-to-head comparisons lacking, but also general literature for many of the more recently marketed techniques. For this reason, there are few tools available to assess a change between techniques and therefore a manuscript of these characteristics was necessary. Other limitations in the field of allergology, which make it difficult to evaluate the most appropriate *in vitro* sIgE test for each laboratory, are the variability of allergen panels in the case of molecular diagnosis, the lack of standardization of allergens and therefore their reproducibility and the lack of quality controls [67], [68].

## Conclusions

Currently, several different tests are available *in vitro* determination of sIgE. It is important to know the particularities of each assay in order to establish an accurate diagnosis and, thereby, ensure that the allergic patient is managed properly.
Singleplex and multiplex systems are both useful for allergy diagnosis; the selection of one type or another will depend on the complexity of each case and the specific needs of the patient.
Based on the scientific evidence, together with the accumulated experience in routine clinical practice, it is clear that the results of the various commercial *in vitro* diagnostic tests for allergies are not interchangeable; therefore, it is important that clinicians make every effort to consistently use the same sIgE detection assay for diagnosis, treatment, and follow up.
Any change in the *in vitro* diagnostic test will inevitably require a comprehensive validation study involving both the laboratory and the treating clinicians managing the allergic patient.
A change in the testing method may have implications for the management of the allergic patient, and this could potentially have a negative impact on diagnosis, treatment selection, and follow-up, and could also imply an increase in associated costs, both direct and indirect.
A quality diagnostic assay for allergies should show an optimal balance between analytical sensitivity and specificity, enough scientific evidence to support its clinical utility, a broad portfolio of allergens, and cost-effectiveness.
At present, until more data on new testing methods become available, ImmunoCAP is the technique that best meets the quality criteria discussed in the present document. Moreover, the clinical value of ImmunoCAP for the *in vitro* diagnosis of allergy in the general population is supported by a large body of scientific evidence.

## Supplementary Material

Supplementary Material Details

Supplementary Material Details
