# Supplementary material for: VALIDA project: Validation of allergy in vitro diagnostics assays (Tools and recommendations for the assessment of in vitro tests in the diagnosis of allergy)
Source: Adv Lab Med. 2020 Aug 21;1(4):20200051. doi: 10.1515/almed-2020-0051 (PMC10197418; doi:10.1515/almed-2020-0051)
Supplement: Supplementary file 1 — Supplementary Material Details [file j_almed-2020-0051_suppl_001.docx]

**Supplementary material**

**Table 1.** Completed clinical studies using ImmunoCAP for allergy testing

| NTC Code | Title | Condition | Recruitment (n) | Location |
| --- | --- | --- | --- | --- |
| NCT02328170 | Comparative Study of Specific IgE Levels to Common Foods and Aeroallergens Measured by Euroimmun Allergy and ImmunoCAP | Allergy | 235 | Thailand |
| NCT02666092 | Anisakis Blastocystis Cryptosporidium Fish Serology | Fish allergy | 105 | Francia |
| NCT01264601 | Safe Administration of Flu Vaccine to Egg Allergic Children | Egg allergy | 31 | United States |
| NCT00715156 | Role of Recombinants in Peach Allergy | Food allergy | 148 | Italia |
| NCT01634737 | Crustacean Allergy and Dust Mites Sensitization | Food allergy | 100 | Italia |
| NCT01641731 | Specific Oral Tolerance Induction in Children Allergic to Cow’s Milk Proteins | Milk allergy | 55 | Spain |
| NCT01792232 | Effects of Co-Exposure to Air Pollution and Allergen | Allergy | 18 | Canada |
| NCT02979600 | Clinical and Biological Efficacy of Peanut Oral Immunotherapy | Peanut allergy | 493 | Francia |
| NCT02644785 | Serum Tryptase Levels During Cardiac Surgery, Diagnosis and Treatment Decisions for Allergic Reactions | Protamine allergy | 30 | Turkey |
| NCT01589731 | Polymerized Beta-lactoglobulin Comparative Immunoreactivity | Milk allergy | 114 | Brasil |
| NCT01489553 | Egg Oral Immunotherapy | Egg allergy | 9 | United States |
| NCT00597675 | Oral Immunotherapy for Peanut Allergy (PMIT) | Food hypersensitivity | 10 | United States |
| NCT03048149 | Clinical and Biological Efficacy of Hazelnut Oral Immunotherapy | Allergy | 100 | Francia |
| NCT01552161 | Prevalence of Allergic Diseases and Atopy in Subjects With Coronary Artery Disease | Allergy | 300 | Poland |
| NCT02382718 | FAST Fish Phase IIb Clinical Trial for the Treatment of Fish Allergy by Subcutaneous Immunotherapy | Fish allergy | 45 | Denmark, Greece, Iceland, Netherlands, Poland, Spain |
| NCT01007253 | Effect of Veramyst and Olopatadine 0.2% Ophthalmic Solution on Allergy Symptoms | Seasonal allergic rhinitis | 21 | United States |
| NCT01966224 | A Safety and Immunogenicity Phase IB Study of CryJ2-DNA-Lysosomal Associated Membrane Protein (CryJ2-DNA-LAMP) Plasmid Assessing the Long-Term Safety of Previously Treated Subjects | Allergic rhino-conjunctivitis | 17 | United States |
| NCT02486159 | The Oligonucleotide Chip Analysis for Allergic Rhinitis Treatment in Herbal Plaster and Acupuncture | Rhinitis | 50 | China |
| NCT00346398 | Promoting Tolerance to Common Allergens in High-Risk Children: Global Prevention of Asthma in Children (GPAC) Study | Asthma | 51 | United States and Australia |
| NCT01644617 | A Dose-Ranging Study of the Safety and Effectiveness of MK-8237 in the Treatment of House Dust Mite (HDM) Induced Allergic Rhinitis/Rhinoconjunctivitis in Adults (MK-8237-003/P07627) | Perennial allergic rhinitis | 124 | Austria |
| NCT02733016 | Seinäjoki Adult Asthma Study | Asthma | 259 | Finland |
| NCT01942096 | Study of Airway Inflammation in Relation to Exercise in Elite Athletes | Exercise–induced asthma | 66 | Belgium |
