# Supplementary material for: VALIDA project: Validation of allergy in vitro diagnostics assays (Tools and recommendations for the assessment of in vitro tests in the diagnosis of allergy)
Source: Adv Lab Med. 2020 Aug 21;1(4):20200051. doi: 10.1515/almed-2020-0051 (PMC10197418; doi:10.1515/almed-2020-0051)
Supplement: Supplementary file 2 — Supplementary Material Details [file j_almed-2020-0051_suppl_002.docx]

**Table 2.** Completed interventional studies with ImmunoCAP (Thermo Fisher)

| Allergy | NTC Code | (n) total | Location |
| --- | --- | --- | --- |
| Egg | NCT01264601 | 31 | United States |
| Allergic rhinitis | NCT02146781 | 22 | United States |
| Rhino-conjunctivitis | NCT01966224 | 17 | United States |
| Allergic rhinitis | NCT02486159 | 50 | Taiwan |
| Sensitivity to cow milk proteins | NCT01641731 | 55 | Spain |
| Allergy | NCT01792232 | 18 | Canada |
| Asthma | NCT00346398 | 51 | United States/Australia |
| Food hypersensitivity | NCT00597675 | 10 | United States |
| Allergic rhinitis (hay fever) | NCT01007253 | 21 | United States |
| Perennial allergic rhinitis | NCT01644617 | 124 | Austria |
| Fish allergy | NCT02382718 | 45 | Denmark, Greece, Iceland, Netherlands, Poland, Spain |
